# Austrian nurses’ positive opinions on geriatric care and their ideas for tackling challenges in caring for the ageing population– a modified focus group study in long-term care

**DOI:** 10.1186/s12912-025-03793-4

**Published:** 2025-09-01

**Authors:** Lena Maria Lampersberger, Eva Pichler, Christa Lohrmann, Franziska Großschädl

**Affiliations:** Institute of Nursing Science, Medical University of Graz, Graz, Austria

**Keywords:** Long-term care, Geriatric nursing, Ageing population, Nominal group technique, Freelisting interviews

## Abstract

**Background:**

As the demand for long-term care increases due to an ageing population, ensuring continuous and high-quality geriatric nursing is crucial. However, geriatric nursing faces challenges such as workforce shortages, high workload, and high emotional demand, which can impact quality of care, job satisfaction, and career change. Little is known about nurses’ opinions on geriatric nursing and their ideas for addressing challenges in caring for the ageing population. This study aims to explore what nurses in Austria value about working in geriatric nursing and their ideas about addressing challenges in geriatric nursing.

**Methods:**

A descriptive qualitative study was conducted using a modified focus group approach that included freelisting interviews and the nominal group technique. The two used sampling methods (i.e., convenience and snowball sampling) generated a convenience sample of 12 participants. Two focus group workshops were held in 2025 with nurses working in long-term care in Austria. The freelisting data were analysed using salience analysis. The ranked ideas generated in the nominal group technique were analysed using Van Breda’s method of ranking.

**Results:**

Nurses valued the appreciation they received from colleagues and older persons they care for, the opportunity to work with the family and relatives of older persons, and their ability to provide individualised and person-centred care. A skilled geriatric nurse was described as possessing empathy, sensitivity, and broad expertise in health care. The three highest-ranked ideas for strengthening geriatric nursing were (1) Promoting/strengthening older persons’ self-care abilities (e.g., providing holistic, individualised care), (2) Coordinating care between services for the older persons, and (3) Promoting/strengthening the team.

**Conclusions:**

These explorative findings suggest that strengthening geriatric nursing requires structural attention to improvements such as reducing bureaucracy, enhanced collaboration across healthcare sectors with the assistance of community nurses, and recognition of nurses’ competencies using a skill- and grade-mix. Implementing a supportive work environment (e.g., adequate equipment/staff/time), optimising skill- and grade-mix approaches, and listening to nurses’ expert insights may contribute to sustainable and high-quality long-term care for the ageing population. Future research should consider a co-research design to plan and introduce target group-specific interventions in geriatric nursing based on nurses’ suggestions.

**Clinical trial number:**

Not applicable.

**Supplementary Information:**

The online version contains supplementary material available at 10.1186/s12912-025-03793-4.

## Background

‘‘*Caring for older people can be challenging*,* beautiful*,* and rewarding’ […] (QN 103*,* 34 years*,* long-term care)*’ [1, p. 8] a nurse wrote about the care of older people in a study assessing nurses’ experiences and opinions on caring for older people [1]. Although this quote reflects a positive opinion about geriatric nursing, an international body of research shows that the career choice of geriatric nursing is not a popular one among nursing students and nurses [2–4]. For example, only 14% of newly registered Canadian nurses had the intention to work in geriatric care [3]. In a Norwegian study, half of the nurses working in geriatric care were thinking about or had the intention of quitting their job [5]. In a US-American study, less than one third of geriatric nurses would recommend their career to others [6]. The global nursing community is currently facing a shortage in personnel, and it is estimated that by 2030, there will be a shortfall of 4.5 million nurses [7, 8], a situation compounded also by the demographic shift towards an ageing population [9]. However, it is predicted that there will be a greater demand for well-trained and specialised geriatric nurses to ensure the quality of nursing care for older people with a need for care due to higher rates of care dependency and care needs [8, 10, 11].

When a person’s need for care arises, holistic nursing interventions must be taken in an adequately equipped care system to ensure that people in need of care receive appropriate and timely care to meet their physical, mental, social, and spiritual needs. Holistic nursing interventions focus on these physical, psychological, sociological, and spiritual needs of a person rather than on their disease or care need [12]. The need for holistic, integrated care of the ageing population is often met within the long-term care sector [13]. The WHO (World Health Organization) defines long-term care as activities ‘*to ensure that people with or at risk of a significant ongoing loss of intrinsic capacity can maintain a level of functional ability consistent with their basic rights*,* fundamental freedoms and human dignity’* [13, p. 7]. Long-term care aims to provide care for people who need assistance with activities of daily living to maintain functional ability [13]. It focuses on the health, personal care, and social needs of people in care [13]. In the field of long-term care, the demographic of service users is mostly composed of older people where, frequently, assistance is needed with activities of daily living [14, 15].

This care can be provided in a variety of settings, including the person’s home, the community, or nursing homes [13]. Depending on the level of care needed, older people prefer to stay in their own homes or in familiar surroundings if they have moderate care needs, but prefer residential care if they have greater care needs [16]. To ensure that long-term care is sufficient and sustainable, sufficient numbers of well-trained nurses are needed in this setting to provide appropriate, high-quality care in the right setting for each individual [9].

Nursing professionals, including qualified nurses and nursing assistants, make up the majority of the long-term care workforce besides informal caregivers [7, 8]. According to Eurofound [8], it is concerning that, in Austria, three out of four people working in acute or long-term care have considered leaving their position, as it is estimated that, in future, 5–10% more qualified nurses will be needed in long-term care. Working conditions (physical workload, mental workload, and time pressure) have an impact on the willingness to continue working in long-term care [17].

Looking at the working conditions prevalent in European long-term care, there are several points of criticism voiced by nurses, such as low pay, low quality of working time (e.g., part-time work, shift work, and working at short notice), high emotional demands [8], and a high risk of experiencing forms of violence (e.g., verbal or sexual harassment or physical violence) [8, 18]. In a study conducted among Austrian nurses, it was found that working conditions (e.g., lack of resources to provide quality care, consequences for physical and mental health) were criticised more often than the geriatric nursing profession itself [1]. These factors increase the exposure of nurses to the risks of impaired physical and mental health, occupational accidents, cardiovascular diseases, burn-out, depression, injuries, lower job satisfaction, and higher employee turnover rates [8, 18, 19]. Furthermore, due to these factors, nurses have the feeling of not being able to provide sufficient care to improve the quality of life of those being cared for due to, for example, lack of time [20]. In a Swedish study, nurses perceived geriatric nursing positively as holistic and respectful work where they could build long-term relationships with the persons they care for and their families and provide individualised and person-centred care [21]. These positive opinions of nurses on geriatric nursing influence their intentions to embark on a career in geriatric nursing or to stay in the profession [3, 6, 22, 23], which might be explained by the Theory of Reasoned Action [24].

Fishbein and Ajzen’s [24] Theory of Reasoned Action shows that opinions are formed by experiences, by interactions with a person, or by information from an outside source. By forming these opinions, one links attributes to an object (e.g., geriatric nursing) which are evaluated (positively, negatively, or neutrally) and lead to the formation of attitudes (e.g., towards geriatric nursing). The sum of opinions and attitudes towards something then influences one’s intention to perform a specific behaviour (e.g. to not consider a career in geriatric nursing, to leave the profession) [24]. Since Covid-19, the pandemic has taken a toll on health care and on long-term care, and the nursing shortage has intensified [25]. Nurses’ opinions about long-term care may now be more critical and negative, as this work has become more demanding and nurses have repeatedly been criticising the work environment, meaning that their interactions and experiences with geriatric nursing is more strained [1, 20]. In the international literature, there is a lack of recent research on long-term care nurses’ perceptions of long-term care and on what strengths they see in their work. Furthermore, the voice of nurses regarding what they need to provide care in a way that they, as experts, consider to be of high quality is rarely heard [1, 20]. In the light of a globally ageing population and nursing staff shortages, this knowledge might be needed in order to plan target groups and implement specific interventions to support geriatric care in a way that is meaningful and needed by those who are affected [25]. Therefore, in this study, we aim to investigate nurses’ priorities with regard to two research questions:

1. What do nurses value about working in geriatric care?
2. What are nurses’ ideas on how to address challenges in geriatric care?

## Methods

This study is a descriptive qualitative study using a modified focus group method including freelisting interviews [26] and the nominal group technique [27]. These methods are especially suitable for target groups with a high workload and lack of time resources [26, 28]. We therefore deemed these methods suitable for focus groups including nurses who work in a stressful and understaffed environment [25]. In addition, freelisting interviews can reveal the priorities and attitudes of a culture [26], and the nominal group technique is particularly useful for focus groups where the goal is to generate ideas and find consensus on their priorities [27, 28]. The Standards for Reporting Qualitative Research (SRQR) checklist was used to guide the reporting of this study [29].

### Setting and Participants

In this study, long-term care is defined as care provided by nurses in a long-term care facility, at home, or in the community, including nursing homes, home care, and community care. In Austria, home care nurses provide professional care in the home of the person they care for, whereas community nurses work preventively and aim to recognise and reduce the need for care at an early stage. They provide information and guidance to the persons they care for in their homes [30]. Nurses working in long-term care in southern Austria were invited to join the focus groups planned as a workshop and entitled ‘What do you wish for? Ideas for strengthening geriatric nursing’. Nurses with a diploma, specialised nurses (i.e., with a Bachelor degree or a diploma), or nurses without a diploma who actively worked in geriatric nursing were invited to participate. In Austria, nurses earn either a Bachelor degree or a diploma after completing a three-year training programme. After graduating, they can specialise in areas such as intensive or palliative care by completing a one-year university course. Nurses without a diploma can specialise by attending training programmes lasting one or two years. Since all these nursing professions are involved in geriatric nursing [31], they were included in this study.

### Sampling

Two sampling methods were used to recruit participants for this study: (1) convenience sampling and (2) snowball sampling. (1) At the beginning of January 2025, an invitation to participate in the workshop and a flyer including information about the workshop were mailed to all residential and home care facilities, to nursing managers in these facilities, and to community nurses in a city in southern Austria and the surrounding area. A list of mail addresses was retrieved from government homepages [32–35]. The nurses were asked to forward the invitation and flyer to their network and to other nurses who might be interested in participating. Reminders were sent out two weeks later. Invitations were sent only to institutions in this particular city and its surrounding areas, as the workshop was held at the local medical university and to ensure short commutes for participants. The flyer was also shared on social media platforms (LinkedIn and Instagram) at the beginning of January 2025 and reposted twice in the following month. The local nursing association and local stakeholders also shared the invitation via their social media channels. Use of social media platforms was chosen to increase the possibility of reaching interested nurses who might not have received an invitation. (2) Nurses in the researchers’ network were asked directly if they wanted to participate, were given a flyer and were asked to bring interested colleagues to the workshop. The aim was to recruit participants for two workshops with 6–10 participants each because, according to the literature, this is the ideal group size for conducting meaningful discussions within the scope of the nominal group technique [36].

In total, 12 nurses participated in the two workshops. They had a median age of 34.5 years and a median geriatric working experience of 9 years. The demographic and professional characteristics of these participants can be found in Table 1.

**Table 1:** Demographic and professional characteristics of participants (*N* = 12)

Variable	Category	*n*
Sex	Female	10
	Male	2
Profession	Nurse with a diploma	7
	Nurse without a diploma	5
Setting	Nursing homes	7
	Home and community care	5

### Focus groups

We conducted two focus groups between February 11th and 13th, 2025. The focus group was designed as a workshop for nurses to work on ideas regarding challenges in geriatric nursing. Two researchers facilitated the workshop, with one (LML) leading the workshop and the other (EP) taking notes, making audio recordings of discussion phases, and preparing data from the freelisting interviews and the nominal group technique for presentation to the participants at the end of the workshop.

**Fig. 1:**
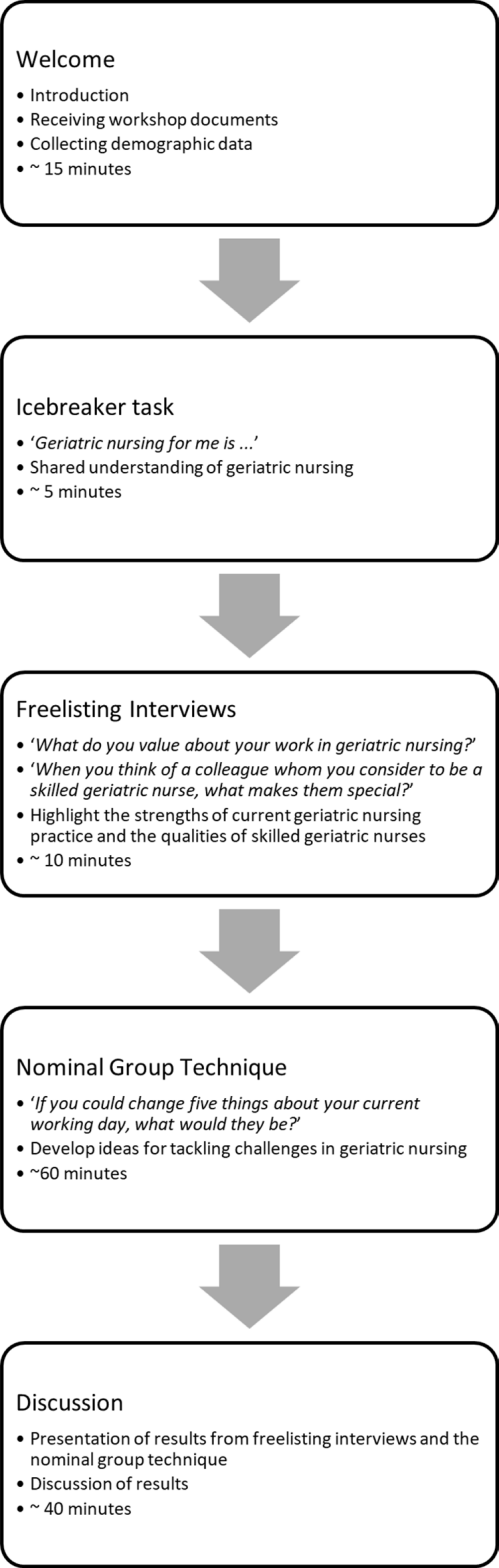
Overview of the five phases of the workshop

Each workshop consisted of five phases and lasted for three hours: (1) welcoming the participants and collecting demographic data, (2) an icebreaker task to get the participants into the mood for the topic and to create a basis for discussion, (3) two freelisting interview questions to assess positive opinions on geriatric nursing and what qualities they attribute to a skilled geriatric nurse, (4) collecting and prioritising ideas for tackling challenges in geriatric nursing using the nominal group technique, and (5) discussing the results. Figure 1 shows an overview of the five phases of the workshop, which are also described in detail below. See Additional File 1 for the workshop guide and demographic questionnaire used.

1. At the beginning of the workshop, participants were welcomed by the researchers, and the researchers and the participants introduced themselves. Each participant was handed a package of materials that included the written informed consent, a questionnaire on demographic data, sheets for freelisting, and sheets for the nominal group technique. Demographic data included sex and age of participants, followed by questions about their work. They were asked about their profession, the setting in which they work, and the number of years they have been working in geriatric nursing. This took about 15 min.
2. Next, the workshop leader explained the steps included in the workshop and gave a brief overview on what the workshop was about. As an icebreaker, participants thought about how they would complete this sentence: ‘*Geriatric nursing for me is.*’. They were then asked to share their understanding of geriatric nursing with the group, if they were willing to do so. Five minutes were scheduled for this phase.
3. Two questions were worked on with the freelisting interview technique. The first was ‘*What do you value about your work in geriatric nursing?*’, and the second was ‘*When you think of a colleague whom you consider to be a skilled geriatric nurse*,* what makes them special?*’ Participants were asked to list anything that came to mind on a prepared sheet. They were given 5 min per question. If participants were still listing items after five minutes, they were given more time. Freelisting interviews identify items in a cultural domain or emic category and show what is most important or salient to the participants, as they tend to list familiar items first [26]. In total, the freelisting interviews took 10 min.
4. In the nominal group technique phase, the participants focused on one question: ‘*If you could change five things about your current working day*,* what would they be?*’. The nominal group technique has four stages [27]: (1) The first stage, ‘idea generation’, consists of each participant writing down ideas individually, one idea per card. In our workshop, participants had to write down five ideas each and were given 10 min. (2) The second stage is called ‘round robin’, where participants state their ideas one by one and stick the notes with the ideas on a flipchart. In this stage, the ideas were not discussed or clarified. 15 min were allotted for this stage. (3) The third stage was a group discussion of the generated ideas. In this stage, ideas could be clarified, discussed, grouped or further divided until a consensus was reached in the group. In addition, an audio recording was made of the discussion. The workshop leader recorded the agreed ideas and their explanations on a whiteboard (ca. 30 min). (4) Finally, in the fourth stage, participants voted on their top three ideas and ranked them using a prepared sheet. This stage lasted 5 min. In total, one hour was scheduled for the nominal group technique.
5. At the end of the workshop, the results of the freelisting interviews and the nominal group technique were presented to and discussed with the participants. An audio recording was also made of this discussion (ca. 40 min).

### Data processing and analysis

Like the focus groups, the analysis also consisted of several stages. First, demographic data were analysed using descriptive statistics in Microsoft Excel (Microsoft Office LTSC Professional Plus 2021) [37]. Notes of the icebreaker task were entered into the software MAXQDA 2020 [38].

Second, freelists were entered into Microsoft Excel (Microsoft Office LTSC Professional Plus 2021) [37] by the researcher who had led the focus groups. The participants and two groups were pooled, and synonymous items were grouped into one item. This process was checked for accuracy by a second researcher who had also been part of the focus group. This resulted in one list per question. A salience analysis was performed on these two lists. This involved calculating the salience of each item on each participant’s list. The ranked items were numbered inversely and divided by the total number of items. The composite salience value was then calculated by dividing the sum of salience per item by the number of participants. There are no clear boundaries for what value is considered salient, thus we determined the salience of items according to breaks in the data visualised by bar graphs (see Additional File 2 and Additional File 3) [26]. It is described by Quinlan [26] that determining the salience of items is a *‘matter of judgement’*, as salience is relative. Usually, visible breaks in the data occur. The first one occurs after those highly salient items which many participants mention at the beginning of their list, the second one after the somewhat salient items which still a lot of participants wrote down high on their lists. Items after these brakes are not considered salient. In addition, the salience of items is also determined by taking into account the frequency of items on all lists.

Third, the nominal group technique was analysed integrating both groups according to the method proposed by Van Breda [39]. The method by Van Breda [39] is designed to incorporate data from more than one nominal group technique into a single analysis and combines qualitative (content analysis) and quantitative (ranking) analysis methods. The generated ideas were entered into Microsoft Excel [37] spreadsheets, and the top five ideas were identified by average scores. Each first-ranked idea received 15 points, second-ranked ideas received 10 points, and third-ranked ideas received 5 points. A content analysis of the generated ideas was then performed in MAXQDA 2020 [38], and overarching themes of ideas were defined. The content analysis was performed by one author and peer reviewed by a second author, both of which had conducted the workshops. Finally, the combined ranks of both groups were calculated by a ‘top 5 score’ that includes only rated ideas, a ‘number score’ that includes the frequency of themes, and an ‘average score’ that standardises the score of ideas independently of group size [39]. The audio-recorded discussions were transcribed using NoScribe (version 0.6) [40] and checked for accuracy by one of the researchers. The analysis was conducted in the original language of the workshops (German). Quotes included in the results were translated into English by one author and DeepL Translator (Version 24.11.4.14424) [41] and checked by a bilingual English native speaker.

### Ethical considerations

This study was approved by the Ethics Committee of the Medical University of Graz, Austria (EK Number 31–320 ex 18/19). Participants signed a written informed consent and were informed that they could withdraw from the study at any time. Digital data is stored on the university’s secure network, and written data is stored securely at the university. Only the researchers have access to the data. This study was conducted in compliance with the Declaration of Helsinki.

### Rigour

To enhance trustworthiness and credibility, the two researchers (LML and EP) that were involved in conducting the workshops met beforehand to discuss the workshop guide in detail and to reflect on their possible influence on the process and on participants. To ensure authenticity, both researchers are nurses with a diploma and experienced in the geriatric setting. Following the first workshop, the understandability of methods and questions were evaluated with the participants and discussed by the two researchers. No changes were made. After each workshop, peer debriefing was carried out during which the researchers reflected again on possible influences they might have had on the discussions and on group dynamics. To ensure reliability and validity of the data, member checks were carried out by presenting results of the workshop to the participants at the end of the workshop. The participants had the opportunity to discuss the results, confirm their validity, and to add additional remarks. The data analysis was performed by one author and peer reviewed by a second author. If necessary, discrepancies were discussed until a consensus was reached. Every step of analysis was discussed by the two authors involved in the analysis process [42].

## Results

The workshops began with nurses reflecting on what geriatric nursing means to them. After this initial icebreaker exercise, the workshop moved on to the freelisting interviews. In response to the first workshop question *‘What do you value about your work in geriatric nursing’*, the most salient item was that nurses experience a lot of gratitude or appreciation for their work, either from their colleagues or from the people they care for. Nurses also have a positive opinion regarding the opportunity to support and challenge the older person they care for and their self-care abilities. This was followed by the opportunity to work with the older person they care for, their families, and their relatives. All salient items generated by the nurses in relation to valuing geriatric nursing can be found in Table 2, and all generated items can be found in Additional File 2 and 4. Additional File 4 includes the frequency and composite salience of all generated items.

**Table 2:** Freelist of salient items of nurses’ positive opinions on geriatric nursing (*N* = 12)

‘What do you value about your work in geriatric nursing?’	Frequency	Composite salience
Lots of gratitude/appreciation (persons they care for/colleagues)	9	0.46
Supporting and challenging older persons and their care needs	4	0.26
Working with the family and relatives of older persons	5	0.23
Teamwork/support/help within the team	5	0.22
Wide-ranging/complex field of activity	3	0.21
Providing support and involvement in the everyday life of the older person	4	0.20
Autonomy/taking responsibility	5	0.19
Personalised care planning according to needs/assessment of needs	5	0.19
Communication/listening/talking/laughing/crying together	4	0.18
Enabling years of healthy life/quality of life	3	0.17
Working in a multi-professional team, utilising networks and interprofessional approaches	5	0.17
Working with people	2	0.16

Note: Higher composite salience scores indicate higher salience; composite salience is calculated by inversely numbering the rankings per list per participant and dividing them by the total number of items per list. The composite salience value is then calculated by dividing the sum of salience per item by the number of participants on a combined list per question [26]

After the second freelisting activity, the nurses collected items in response to the second workshop question: *‘When you think of a colleague whom you consider to be a skilled geriatric nurse*,* what makes them special?*’ The most salient point was that a skilled nurse possesses a high degree of empathy, sensitivity, and compassion. A skilled nurse also requires expertise and broad knowledge in health care, and takes time for the people in their care, even when under stress. All salient items that the nurses attributed to a skilled geriatric nurse can be found in Table 3, and all generated items and their composite salience can be found in Additional File 3 and 4.

**Table 3:** Freelist of salient items of what nurses consider to be a skilled geriatric nurse (*N* = 12)

‘When you think of a colleague whom you consider to be a skilled geriatric nurse, what makes them special?’	Frequency	Composite salience
Has a high degree of empathy/sensitivity/compassion	11	0,65
Has expertise and knowledge in health care	7	0,31
Takes their time (despite stress)	4	0,30
Is very organised/structured/precise	5	0,30
Has several years of professional experience	4	0,26
Has patience/a calm disposition	5	0,23
Listens closely/has an open ear	5	0,22
Is constantly learning, regularly participates in ongoing training	4	0,18
Shares knowledge/guides other employees/helps colleagues	5	0,16
Respects individual wishes	3	0,16
Is caring/has a gentle manner with older persons	2	0,15
Works with foresight, keeps sight of what’s important/able to assess situations	3	0,14
Is motivated	2	0,14
Has a sense of humour	4	0,14
Is reliable	2	0,14
Is able to observe and perceive well	2	0,13
Prioritises taking into account the environment of older persons	3	0,13
Can express themself well/ has good communication skills	6	0,13

Note: Higher composite salience scores indicate higher salience; composite salience is calculated by inversely numbering the rankings per list per participant and dividing them by the total number of items per list. The composite salience value is then calculated by dividing the sum of salience per item by the number of participants on a combined list per question [25]

After the nurses had discussed positive opinions on geriatric nursing and the attributes they assign to a capable geriatric nurse, the participants used the nominal group technique to develop their own ideas for strengthening geriatric nursing. This was guided by the third workshop question ‘*If you could change five things about your current working day*,* what would they be?*’

After the individual ideas were discussed in the groups and the researchers analysed the themes of the ideas collected and agreed upon, eight themes of ideas emerged (see Table 4). The three ideas that where most important to the participants and therefore ranked higher were (1) ‘Promoting and strengthening older persons’ self-care abilities’, (2) ‘Coordinating care for the older persons between services’, and (3) ‘Promoting and strengthening the team’.

**Table 4:** Ranking of generated ideas for strengthening geriatric nursing using the analysing method by Van Breda [39]

Themes of ideas	Top 5 votes	Average score	Final rank score
**Promoting and strengthening older persons’ self-care abilities**	**3**	**33.75**	**23**
**Coordinating care for the older persons between services**	**2**	**30**	**18.5**
**Promoting and strengthening the team**	**2**	**20**	**15.5**
Strengthening the image and value of geriatric nursing	1	25	14.5
Facilitating holistic prevention	1	40	14.5
Nursing homes as suitable place for individual old age	1	25	11
Promoting specialised training and professional development	0	15	6
Promoting equal opportunities for persons in care	0	10	5

Note: Higher final rank scores indicate higher ranking of ideas; the top three ranked themes of ideas are presented in bold. Scores were calculated according to Van Breda [39]

### Promoting and strengthening older persons’ Self-Care abilities

Strengthening older persons’ self-care abilities can be achieved, on the one hand, by the older person and, on the other, with the help of nurses, facilities and structures. To be able to provide care that promotes and strengthens the self-care abilities of older persons, participants discussed that holistic care which is tailored to the older person based on their biography was needed. They suggested that early on, when a need for care first arises, persons in care should write down their needs and preferences. A nurse described that *‘[…] for each activity of daily life*,* the person should write down how they would like it to be. For example*,* would they prefer a shower or a bath*,* warmer water or do they like it a little cooler.’* (HCCN 2.1).

With regard to this topic, nurses further discussed that nursing services in particular are not sufficiently publicly funded, which leads to unequal opportunities for persons in care. They said, *‘At the moment*,* it depends on whether I can afford it or not. It’s about equal opportunities.’* (HCCN 1.3). They proposed that home care, family caregivers, community nursing and social interventions should be better funded by public funds. They further discussed that to relieve the burden on family caregivers, it should be possible to receive funding for support provided by nursing professionals for a few hours a day. HCCN 1.3 states the problem as follows: *‘It is often the case that some people need support throughout the day […] That they cannot provide home care for the entire day […] That is not actually subsidised at all. You have to pay for it yourself.’*

Furthermore, participants discussed that in order to provide holistic care in addition to basic nursing interventions, they need more time with the person they care for, i.e., for individual care, social interventions, and preventive interventions. They suggested that more time resources can be made available by reducing bureaucracy and minimising care planning to essential interventions. NH 2.2, for example, proposed that as follows: ‘*I find that many things*,* especially in care planning*,* are no longer all that up-to-date such as the housekeeping components*,* making the bed*,* for example*,* […] so you can cut out a lot of that*,* that’s what I’m saying. So that you don’t need so much time for care planning either.*’ By using technology in the care process (e.g., in planning nursing interventions or when completing documentation) more extensively and in a responsible and meaningful way, the participants believe that more time resources can be made available. They emphasized that it is important to receive training in the use of technology in order for them to make efficient use of it.

Another possibility to strengthen older persons’ self-care abilities is involving their relatives, as they can provide support in the care process. Participants promoted *‘[…] training of relatives in nursing activities’* (RLTC 2.2) and see community nurses as an opportunity to strengthen the relatives’ skills in nursing, and see a possible impact on personnel shortages. Nurses also discussed that with less family members involved in care nowadays, there is also a shortage of relatives, which makes it difficult to integrate relatives into care and can put people in care without relatives at a disadvantage, which led them to the theme of the idea ranked second: to ‘*coordinate care for the older person between services*’.

### Coordinate care for the older person between services

The second highest ranked idea was that the care an older person receives needs to be better coordinated between different institutions and settings. Participants noted that *‘[…] there are already a lot of organisations and hospitals that are jumping on the bandwagon [coordinating intra- and extramural care]’* (HCCN 1.4). Even though they see lighthouse projects making initial efforts, they see opportunities to further strengthen the care of older people. They said, *‘Many people are no longer mobile and can’t go to their GP [general practitioner]*,* but they [GPs] don’t make home visits. They actually fall completely through the cracks if there is no one to make home visits.’* (HCCN 1.4). The participants said that community nurses would be a great resource to strengthen care at home and to coordinate between different institutions and settings. For example, HCCN 1.5 says, ‘*It’s not just the home visits that are needed*,* it’s making contact while the patient is still in hospital. That I go there as a CN [community nurse].’* Community nurses accompanying the older person from the acute setting to the home setting can be a resource for persons in care to help minimise the feeling of being overwhelmed when coming home from hospital with new care needs. The participants think that community nurses should then explain everything to the patient again (e.g., diagnoses) and educate the older person receiving care and their family on care needs (e.g., leaving the hospital with a urinal catheter or wound they have to take care of at home) if needed according to their care needs. They stressed that planning their care at home should already start (case and care management) upon admission to the acute care setting.

Another way to facilitate this process is to have all institutions, professions, and settings use the same information system, so that, for example, nurses working in the community or in a person’s home and the person being cared for have access to all the information they need. Nurses find it difficult to communicate and share information efficiently; they said, *‘In principle*,* similar things are done [by different professions] and often what is actually needed at that moment isn’t being done.’* (HCCN 1.3). That led participants to their third ranked theme of ideas, promoting and strengthening the interprofessional team.

### Promoting and strengthening the team

The team, mostly consisting of physicians, nurses and nursing assistants, should, according to the participants, work on an equal footing and should always focus on the best interests of the persons in care. RLTC 1.1 said, *‘Because I believe that if the team functions harmoniously within itself*,* then it also responds better to the older person.’* To achieve this goal, nurses proposed strengthening flat hierarchies with good communication skills and a positive work culture in the interprofessional team and in the nursing profession itself. The key is to utilize a person’s skills and to promote a skill- and grade-mix in nursing. ‘*Actually*,* it’s about making use of all competences of every professional group anyway*,* isn’t it? […] I mean above all this skill grade mix. Making full use of it all.’*, says RLTC 2.2.

Further to these top three ideas (see Table 4), nurses discussed their desire to strengthen the image and value of geriatric nursing by giving nurses more competencies and more opportunities for academic and continuous education, and by speaking publicly about the strengths and positives aspects of geriatric nursing (e.g., job rotations, or showing the public how diversified the field of geriatric nursing can be). Furthermore, nurses discussed the necessity of early preventive and personalised interventions to promote a healthy life for as long as possible and therefore wished to strengthen health promotion at schools. When older persons move to a nursing home, nurses suggested that the facility be more careful when accepting new residents. They also suggested that nurses should be consulted to ensure that the nursing home is the right place for each person’s individual care needs, as they have more expertise in assessing the needs of the older person. Within the theme ‘*promoting focused training and professional development’*, nurses suggested that more focused geriatric continuous education should be offered to nurses with and without a diploma. Furthermore, they suggested that nurses should be able to attend courses at nurses’ undergraduate education facilities so that they can refresh and update their knowledge and students can learn from their experience. Lastly, nurses insisted that persons in care should receive equal opportunities independently of their wealth, network, and amount of family support.

In the final discussion of the workshops, nurses reflected positively on their ideas and said that it is their goal to provide best possible care, *‘and the only way to do that is to interlink the care services so that the person*,* so to speak*,* with the clinical presentation*,* can be treated individually*,* despite the diversity.’* (HCCN 1.3).

## Discussion

This study asked two research questions: (1) What do nurses value about working in geriatric care?, and (2) What are nurses’ ideas on how to address challenges in geriatric care? Nurses appreciate the gratitude they receive from those they care for and from their colleagues. They also value the fact that they are able to support and challenge the older person they care for and their self-care abilities. The participants also attributed empathy, sensitivity, and compassion to a skilled geriatric nurse. They also value the expertise and knowledge in health care a geriatric nurse holds. In regard to the second question, three ideas for strengthening geriatric nursing were highlighted by the participants: (1) *‘Promoting and strengthening older persons’ self-care abilities*, (2) *‘Coordinating care for the older persons between institutions’*, and (3) *‘Promoting and strengthening the interprofessional team’*.

In a previous study conducted in Sweden, nurses stated that what they valued about geriatric nursing was that it has a holistic approach to care, that nurses are able to build long-term relationships with people in care and their families, and that they are able to deliver individualised and person-centred care [21]. Person-centred care means that the values and preferences of the person in receipt of care guide the care process, aiming for realistic life and health goals [43]. By providing holistic care that takes into account the physical, psychological, sociological, and spiritual needs of a person [12], nurses appreciate being able to develop and use more than technical skills, which makes long-term care more complex than acute care [21]. The results of this study led to similar findings, as participants also highly appreciated working with the family and relatives of older persons. Even though they also valued individualised and person-centred care as well as the complexity and diversity of geriatric nursing saliently, it was not ranked top of the list, making the gratitude they receive by colleagues and persons in care more important to them. Receiving gratitude is closely related to the extent to which nurses are satisfied with the care they provide [44]. Furthermore [45], receiving gratefulness and being grateful (e.g., being grateful to your colleague) positively influences job satisfaction by supporting resilience in stressful situations [45, 46]. It may therefore have an impact on staying in the profession, as job satisfaction is linked to turn-over rates [47].

This might be linked to Fishbein and Ajzen’s [24] Theory of Reasoned Action. Amongst others, experiencing gratefulness and receiving gratefulness might lead nurses to form positive opinions about geriatric nursing, which might then lead to greater job satisfaction and lower intentions to leave the profession [48–50]. Furthermore, an Austrian study showed that nurses’ positive experiences with older persons in care had a positive influence on their attitudes towards older people as well as on their perspectives on geriatric nursing [51].

A skilled nurse was described by the participants as having a high degree of empathy, sensitivity, and compassion and as possessing expertise and knowledge in health care. Dierckx de Casterlé et al. [52] describe, in their concept of ‘skilled companionship’, nursing care as bringing together skills (i.e., scientific, nursing knowledge) and companionship (i.e., seeing the person they care for as an individual and being empathetic to their needs). Also, older persons state that these are qualities they would like to see in a skilled nurse, although they rarely experience them. Due to stressful work environments, lack of a caring, patient-centred culture in nursing practice, and lack of teamwork, ‘skilled companionship’ is often not practiced and leads to nurses being dissatisfied with the care they provide [53].

The number one theme relating to ideas for strengthening geriatric nursing was ‘*promoting and strengthening older persons’ self-care abilities.’* A systematic review by Compton et al. [20] of the factors influencing nurses’ or nursing students’ decisions to enter or remain in geriatric nursing shows that nurses’ work is influenced by organisational culture, for example the availability of resources (i.e., staff or equipment). For nurses to be willing to work in geriatric nursing, the work environment and structures in the institutions must be supportive. This means that institutions need to be aware of difficulties in the workplace and provide support. On the other hand, high workloads and insufficient staff discourage nurses from working in geriatric nursing. This led to the feeling of not being able to provide sufficient care to improve the quality of life of those being cared for [25]. Participants proposed integrating greater use of technology into the nursing process, for example when planning nursing interventions or completing documentation. According to international literature, such technologies include, for example, information and communication systems that comprise computerised decision support systems and electronic health records. These are used to collect, store, and manage data of the person and for communication in health care. By using these tools, quality of care and patient safety can be improved, as health-related information is registered and evaluated more clearly, for example to document allergies and medications. Furthermore, the use of this technology can lead to reduced workload, reduced physical and mental pressure, and higher efficiency of nurses [54].

Participants also wished to ‘*coordinate care for the older person between institutions’*, and described the principles of an integrated care system. An integrated care system is a system in which all services needed by an older adult (e.g., home care, acute care, social care, etc.) cooperate in a coordinated way in order to provide person-centred and holistic care [55]. The World Health Organization is working towards achieving an integrated care system in primary health and long-term care by providing a guideline describing key elements of an integrated care system, such as the use of information as well as monitoring and evaluation systems that are linked between services, having well-trained and sufficient healthcare staff, and supporting family care givers [55, 56]. Furthermore, nurses stated that the use of a skill- and grade-mix is needed in nursing practice. Although it is well known from the international literature that a skill- and grade-mix is beneficial for nursing practice and the care provided, participants stated not seeing its full potential being used in daily practice [57, 58].

### Strengths and Limitations

The freelisting interview and nominal group technique methods used in this study proved to be an efficient method for a target group that lacks time resources. Participants reported that the methods were easy for them to use and that they appreciated the process and outcome of both methods. A heterogeneous group of participants enabled us to gain insights into different perspectives and foci of geriatric nursing. The rigour of this study was strengthened through member checks, peer debriefing and peer reviews. This study does, however, have certain limitations. One limitation is that even though we obtained insights into different perspectives using a homogeneous setting, we were not able to portrait the specific suggestions for change prioritised by nurses working in nursing homes and home-care. The context in which they deliver care differs between these two settings. It was, unfortunately, not possible to analyse these groups separately, as the data was not extensive enough. Furthermore, the possibilities with regard to the analysis of the nominal group technique were limited due to the mixed settings of the participants in the two focus groups and because the participants worked together on developing their ideas [59]. Another limitation is that it is recommended to use 20 to 30 study participants in freelisting interviews in order to confidently portrait rankings [60]. In this study, only 12 participants were included, and although the desirable number of participants was achieved for the nominal group technique [36], it was not achieved for freelisting interviews. When interpreting the data of the freelisting interviews, this should therefore be kept in mind. Sampling proved to be difficult, as many nurses declined to participate due to a lack of time resources. A combination of a convenience and snowball sampling method might not have been the best way to obtain a sufficient number of participants, and a purposive sampling method might achieve better results. Nurses with a strong interest in geriatric care may have participated primarily. As this may have introduced a response bias, caution should be used in interpreting these data.

### Implications

In nursing practice, it should be carefully considered which person possesses which competencies, and tasks should be distributed accordingly to get the best out of the skill- and grade-mix in nursing practice and the interprofessional team. Furthermore, fostering a supportive work environment by providing adequate equipment, staff, and time to foster a skill-companionship relationship between the geriatric nurse and the person in their care might lead to higher job satisfaction. It is recommended that policy makers, nursing managers, and researchers give more voice to long-term care nurses, as they work closely with the ageing population, see the needs that exist in practice, and are thus able to contribute important ideas for mastering the challenges in this field. In future research, a co-research design with long-term care nurses could be used to plan and implement an intervention such as the use of technology in the nursing process or interventions to foster a holistic care approach, in accordance with the nurses’ ideas expressed in this study. To be able to identify whether nurses’ positive opinions on geriatric nursing influence job satisfaction and their willingness to stay in the profession in accordance with the Theory of Reasoned Action [24], quantitative studies could be conducted using psychometrically validated scales assessing perception of geriatric care (e.g., the Perspective on Caring for Older People scale [61], the McCloskey/Mueller Satisfaction Scale [62], or the Nurse Turnover Intention Scale [63]).

## Conclusion

This study provides an insight into nurses’ positive opinions on geriatric nursing and what their ideas are for tackling challenges in geriatric nursing. Participants highlighted the importance of receiving gratitude from colleagues and care recipients, of working with the family and with relatives of older persons, and of providing individualised, person-centred care. Nurses identified three ideas for strengthening geriatric nursing: (1) Promoting and strengthening selfcare-abilities of older people, (2) Improving coordination of care across settings, and (3) Improving interprofessional collaboration. Listening to long-term care nurses who have expert insights into the care needs of an ageing population voice their ideas can help in working towards the provision of high-quality geriatric care. Use of a co-research design could be considered, for example, in planning target group- and setting-specific measures in geriatric nursing in accordance with nurses’ ideas on interventions to provide holistic care for tackling challenges in geriatric nursing. In nursing practice, a supportive work environment, which can help address workforce shortages and improve job satisfaction, should be promoted. By working towards these measures, geriatric nursing can be strengthened to provide sustainable, high-quality support for an ageing population.

## Supplementary Information

Below is the link to the electronic supplementary material.

Supplementary Material 1

Supplementary Material 2

Supplementary Material 3

Supplementary Material 4

## Data Availability

The dataset used in the current study is not available because the anonymity of the participants cannot be guaranteed.

## Abbreviations

SRQR: Standards for Reporting Qualitative Research checklist
WHO: World Health Organization
