# Supplementary material for: Austrian nurses’ positive opinions on geriatric care and their ideas for tackling challenges in caring for the ageing population– a modified focus group study in long-term care
Source: BMC Nurs. 2025 Sep 1;24:1139. doi: 10.1186/s12912-025-03793-4 (PMC12400631; doi:10.1186/s12912-025-03793-4)
Supplement: Supplementary file 1 — Supplementary Material 1 [file 12912_2025_3793_MOESM1_ESM.pdf]

Workshop guide

A moderator and a co-moderator will lead the workshop. The facilitator guides the group through the workshop. The task of the co-moderator is to summarize and analyse the data. In addition, she is responsible for ensuring the audio recordings.

The participants sit together on a group of tables, with a buffet set up at the back of the room. At the front of the room there is a flipchart and colourful paper and adhesive strips are ready on a table.

In total, the workshop lasts about 3 hours, start: 14:00

**Welcome**	**20 minutes**
	At the beginning of the workshop, participants will receive a workshop package and a name tag. Participants will be informed about data protection and the study and will be asked to sign the informed consent. The workshop documents will be handed out and the participants will be asked to fill in the socio-demographic data.
**14:15**	Participants are asked to take a seat at the table
	Welcome and introduction of the moderator and co-moderator
	Introduction to the workshop<br><br>In the course of this workshop we want to know from you what you need in the care of older people, what support services you

	would like and what you appreciate about geriatric care. To get closer to this topic, there are two parts in this workshop. After a short round of introductions, in the first part everyone will identify strengths of geriatric care using the freelisting method and in the second part we will explore your wishes using the Nominal Group Technique. I will guide you through all the steps and explain them. At the end of the workshop, we will present the results to you and briefly discuss them with you. But before we begin, I would like to point out that everything that is said in this room is kept confidential. All information collected today is processed anonymously. The discussion rounds are also recorded so that we can analyse them more easily later and incorporate your comments into the analysis. As you can see, all pages of your workshop documents are numbered. These are there so that we can assign all pages to the socio-demographic data without your name appearing. You can opt out of this study at any time.
	Are there any questions from your side?
	Introduction

	We take turns and everyone introduces themselves with their first name, profession and area of work (organizations should not be named) – moderator and co-moderator also participate.
Introduction to the topic	Sit back and think for yourself how you would end this sentence. Geriatric nursing for me is...<br><br>(Give a minute to think about it) What did you have in mind? Does anyone want to complete the sentence out loud?
**Freelisting Interview**	**10 minutes**
	We now start with the first part of the workshop – the freelisting task:<br><br>I will ask you two questions, after the first question you have 5 minutes to write down everything you can think of among yourself. Please use this page in your workshop documents. For example, if I ask the question – What flowers can you think of, then start listing, for example: 1. Daisies, 2. Sunflowers, 3. Roses and so on.

	The first question is:<br>**What do you value about your work in geriatric nursing?**<br><br>The 5 minutes are now over, my colleague is now collecting the page and for the second question please use the next page.<br><br>**When you think of a colleague whom you consider to be a skilled geriatric nurse, what makes them special?'**<br><br>Thank you very much, the 5 minutes are now over. Before we start with the next part, we will take a 10-minute break. Please take something from the buffet.
**10 minute break**	
**Nominal Group Technique**	**1 hour**
**Introduction**	In the next part, we will work out a question using the Nominal Group Technique. This technique is designed to generate ideas together and then prioritize them. This happens in 4 phases. First, you will independently write down 5 ideas regarding the question on these colourful pieces of paper. Then each person in turn says an idea and puts it up on the flipchart. If you come

	up with an addition or further idea, write it down on a piece of paper and keep it in the meantime. You can bring these in the next round. After that, we discuss the ideas, summarize them or split them further and add your additions. If ideas are not entirely clear to you, this can be explained further in this round. This round will also be recorded. In the last round, you will again choose the 3 most important questions for you on your own. Please use this page in your documents and write down the most important idea for you at 1 and so on. Afterwards, we will show you the combined results. Do you have any questions about this? I will explain each step when we do it.
**1. Individual development of ideas**<br><br>10 minutes	We start by writing down the ideas. Each of you writes down 5 ideas.<br><br>The question is:<br>**If you could change five things about your current working day, what would they be?**<br><br>(The question is also clearly visible on the whiteboard/flipchart)
**2. Presentation of the ideas**	We now take turns and everyone presents their ideas for the question. You are only stating the idea now – more detailed

15 minutes	explanations will come in the next round. My colleague sticks the notes on the flipchart. If you then come up with something else or can think of an addition, you will have time to add it later.
**3. Discussion of ideas**<br><br>30 minutes	We are now going through idea by idea. Please explain in more detail what you mean by this, the group can summarize ideas, add to them or contribute new ideas. We take half an hour to work on these ideas. In the end, we should have a clear collection of ideas. This part is recorded. We are starting the recording now.
**4. Vote and prioritize**<br><br>5 minutes	We finish the recording. Now please choose the 3 most important ideas for yourself and enter them on this page. 1 is the most important idea for you. You have 5 minutes to do this.<br><br>My colleague now collects the leaves and we take a 10-minute break
**10 minute break**	
**Presentation of the results**<br><br>10 minutes	In the meantime, the presenter and co-presenter prepare an overview of results.

**Discussion of the results**<br><br>30 minutes	Finally, I ask you to take turns sharing your impressions of the results. What do you think of that? This part is recorded. We are starting the recording now.
**Dismissal**	We have now reached the end of the workshop. Thank you for participating.

Demographic Questionnaire

1. Please indicate your gender

o Female

o Male

o Diverse

o I do not wish to specify

2. How old are you? (in years)

____

3. Which profession do you practise?

o Domestic home help

o Nursing aid (one-year training)

o Nursing aid (two-year training)

o Qualified nurse

o Qualified nurse with specialisation

4. In which setting do you mainly work?

o Residential long-term care

o Home care

o Community nursing

5. How many years have you been working in geriatric nursing?

____
