# Supplementary figures and images for: Austrian nurses’ positive opinions on geriatric care and their ideas for tackling challenges in caring for the ageing population– a modified focus group study in long-term care
Source: BMC Nurs. 2025 Sep 1;24:1139. doi: 10.1186/s12912-025-03793-4 (PMC12400631; doi:10.1186/s12912-025-03793-4)

‘What do you value about your work in geriatric care?’

N = 12

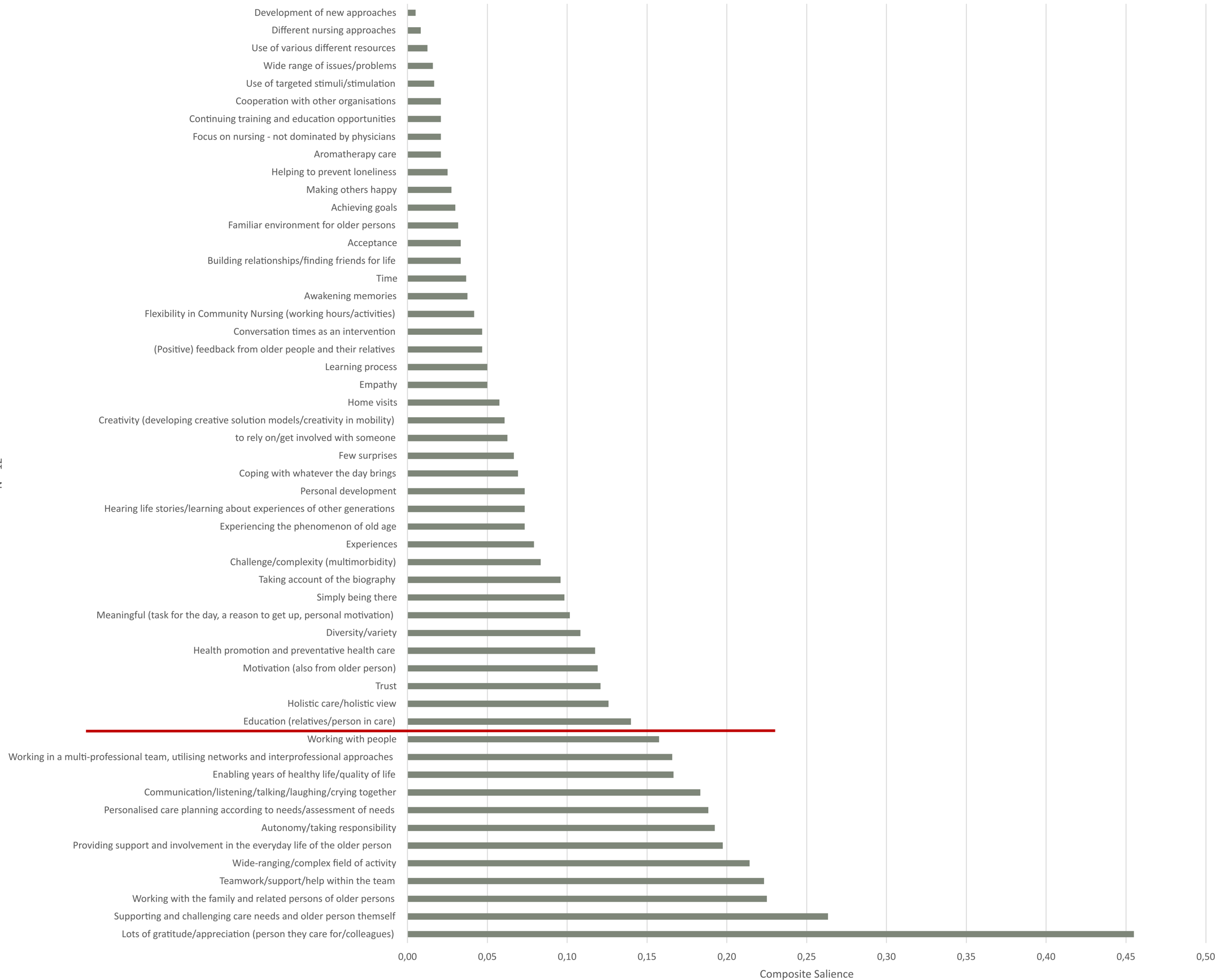

Supplement: Supplementary file 2 — Supplementary Material 2 [file 12912_2025_3793_MOESM2_ESM.pdf]

‘When you think of a colleague you consider to be a good geriatric nurse, what makes them special?’

N = 12

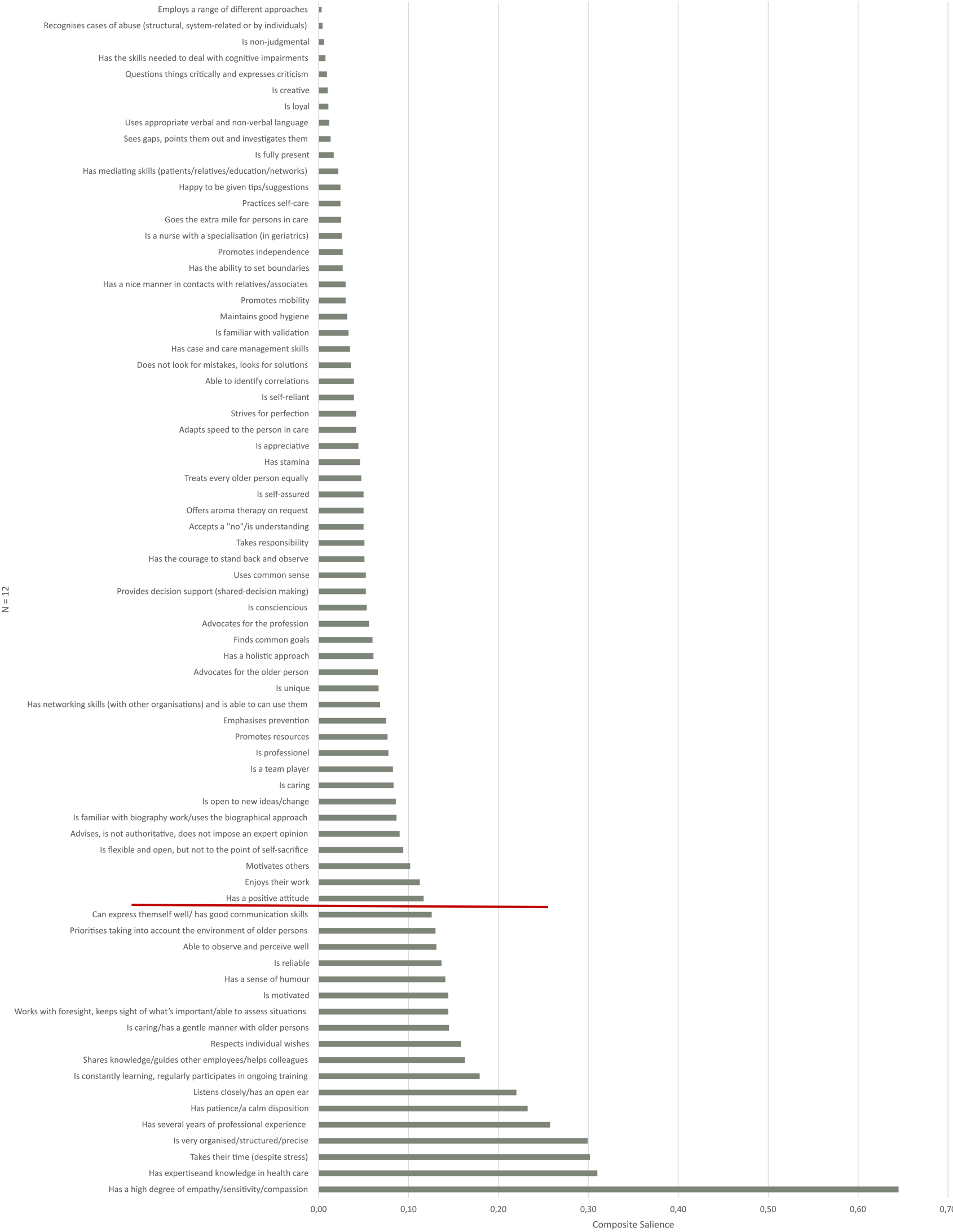

Supplement: Supplementary file 3 — Supplementary Material 3 [file 12912_2025_3793_MOESM3_ESM.pdf]
