# Supplementary material for: Austrian nurses’ positive opinions on geriatric care and their ideas for tackling challenges in caring for the ageing population– a modified focus group study in long-term care
Source: BMC Nurs. 2025 Sep 1;24:1139. doi: 10.1186/s12912-025-03793-4 (PMC12400631; doi:10.1186/s12912-025-03793-4)
Supplement: Supplementary file 4 — Supplementary Material 4 [file 12912_2025_3793_MOESM4_ESM.pdf]

Additional Table 1: Freelist to the question ''*What do you value about your work in geriatric nursing?*'' including all generated items, frequencies and sum salience and composite salience

Item	Frequency	Composite Salience (n=12)
Lots of gratitude/appreciation (person they care for/colleagues)	9	0,46
Supporting and challenging care needs and older person themself	4	0,26
Working with the family and related persons of older persons	5	0,23
Teamwork/support/help within the team	5	0,22
Wide-ranging/complex field of activity	3	0,21
Providing support and involvement in the everyday life of the older person	4	0,20
Autonomy/taking responsibility	5	0,19
Personalised care planning according to needs/assessment of needs	5	0,19
Communication/listening/talking/laughing/crying together	4	0,18
Enabling years of healthy life/quality of life	3	0,17
Working in a multi-professional team, utilising networks and interprofessional approaches	5	0,17
Working with people	2	0,16
Education (relatives/person in care)	3	0,14
Holistic care/holistic view	3	0,13
Trust	2	0,12
Motivation (also from older person)	2	0,12
Health promotion and preventative health care	3	0,12
Diversity/variety	3	0,11
Meaningful (task for the day, a reason to get up, personal motivation)	4	0,10
Simply being there	3	0,10
Taking account of the biography	2	0,10
Challenge/complexity (multimorbidity)	1	0,08
Experiences	1	0,08
Experiencing the phenomenon of old age	1	0,07
Hearing life stories/learning about experiences of other generations	2	0,07

Personal development	1	0,07
Coping with whatever the day brings	1	0,07
Few surprises	1	0,07
to rely on/get involved with someone	1	0,06
Creativity (developing creative solution models/creativity in mobility)	2	0,06
Home visits	1	0,06
Empathy	1	0,05
Learning process	1	0,05
(Positive) feedback from older people and their relatives	2	0,05
Conversation times as an intervention	1	0,05
Flexibility in Community Nursing (working hours/activities)	1	0,04
Awakening memories	1	0,04
Time	1	0,04
Building relationships/finding friends for life	2	0,03
Acceptance	1	0,03
Familiar environment for older persons	1	0,03
Achieving goals	1	0,03
Making others happy	1	0,03
Helping to prevent loneliness	1	0,03
Aromatherapy care	1	0,02
Focus on nursing - not dominated by physicians	1	0,02
Continuing training and education opportunities	1	0,02
Cooperation with other organisations	1	0,02
Use of targeted stimuli/stimulation	1	0,02
Wide range of issues/problems	1	0,02
Use of various different resources	1	0,01
Different nursing approaches	1	0,01
Development of new approaches	1	0,01

Additional Table 2: Freelist to the question '*When you think of a colleague whom you consider to be a skilled geriatric nurse, what makes them special?*' including all generated items, frequencies and sum salience and composite salience

**Item**	**Frequency**	**Composite Salience (n=12)**
Has a high degree of empathy/sensitivity/compassion	11	0,65
Has expertise and knowledge in health care	7	0,31
Takes their time (despite stress)	4	0,30
Is very organised/structured/precise	5	0,30
Has several years of professional experience	4	0,26
Has patience/a calm disposition	5	0,23
Listens closely/has an open ear	5	0,22
Is constantly learning, regularly participates in ongoing training	4	0,18
Shares knowledge/guides other employees/helps colleagues	5	0,16
Respects individual wishes	3	0,16
Is caring/has a gentle manner with older persons	2	0,15
Works with foresight, keeps sight of what's important/able to assess situations	3	0,14
Is motivated	2	0,14
Has a sense of humour	4	0,14
Is reliable	2	0,14
Able to observe and perceive well	2	0,13
Prioritises taking into account the environment of older persons	3	0,13
Can express themself well/ has good communication skills	6	0,13
Has a positive attitude	2	0,12
Enjoys their work	2	0,11
Motivates others	2	0,10
Is flexible and open, but not to the point of self-sacrifice	3	0,09
Advises, is not authoritative, does not impose an expert opinion	2	0,09
Is familiar with biography work/uses the biographical approach	2	0,09
Is open to new ideas/change	3	0,09

Is caring	1	0,08
Is a team player	2	0,08
Is professional	1	0,08
Promotes resources	1	0,08
Emphasises prevention	2	0,08
Has networking skills (with other organisations) and is able to can use them	3	0,07
Is unique	1	0,07
Advocates for the older person	1	0,07
Has a holistic approach	1	0,06
Finds common goals	1	0,06
Advocates for the profession	1	0,06
Is consciencious	1	0,05
Provides decision support (shared-decision making)	1	0,05
Uses common sense	1	0,05
Has the courage to stand back and observe	2	0,05
Takes responsibility	2	0,05
Accepts a "no"/is understanding	2	0,05
Offers aroma therapy on request	1	0,05
Is self-assured	1	0,05
Treats every older person equally	1	0,05
Has stamina	1	0,05
Is appreciative	1	0,04
Adapts speed to the person in care	1	0,04
Strives for perfection	1	0,04
Is self-reliant	1	0,04
Able to identify correlations	1	0,04
Does not look for mistakes, looks for solutions	1	0,04
Has case and care management skills	1	0,04
Is familiar with validation	1	0,03

Maintains good hygiene	1	0,03
Promotes mobility	1	0,03
Has a nice manner in contacts with relatives/associates	1	0,03
Has the ability to set boundaries	1	0,03
Promotes independence	1	0,03
Is a nurse with a specialisation (in geriatrics)	2	0,03
Goes the extra mile for persons in care	1	0,03
Practices self-care	1	0,02
Happy to be given tips/suggestions	1	0,02
Has mediating skills (patients/relatives/education/networks)	1	0,02
Is fully present	1	0,02
Sees gaps, points them out and investigates them	1	0,01
Uses appropriate verbal and non-verbal language	1	0,01
Is loyal	1	0,01
Is creative	1	0,01
Questions things critically and expresses criticism	1	0,01
Has the skills needed to deal with cognitive impairments	1	0,01
Is non-judgmental	1	0,01
Recognises cases of abuse (structural, system-related or by individuals)	1	0,00
Employs a range of different approaches	1	0,00
